# Oral submucosal fibrosis: a comprehensive review on pathogenesis, diagnosis, therapeutics and computational advances

**DOI:** 10.3389/fcell.2025.1754209

**Published:** 2026-01-22

**Authors:** Chinmay Nitin. Mokal, Mrinmoy Das, Sridhar Hannenhalli, Piyush Agrawal

**Affiliations:** 1 Department of oral and maxillofacial surgery, SRM Kattankulathur Dental College and Hospital, Chennai, India; 2 Division of Medical Research, Research Centre, SRM Medical College and Hospital, SRM Institute of Science and Technology, Chennai, India; 3 Cancer Data Science Laboratory, NCI, NIH, Bethesda, United States

**Keywords:** anti-fibrotic therapeutics, areca (betel) nut, diagnostic biomarker, extracellar matrix (ECM), hypoxia, oral squamos cell carcinoma, oral submucocal fibrosis, TGF-β1/Smad3 signaling pathway

## Abstract

Oral submucosal fibrosis (OSF) is a chronic and progressive fibrosis disease and causes sclerosis in oral mucosal tissue with a higher potential of malignant transformation. It is characterized by excessive production and deposition of extracellular matrix. The major behavioral cause of OSF is chewing areca nut, and the symptoms include severe burning sensation, ulceration, restricted mouth opening, and more. However, despite significant advancements in biochemical and molecular techniques in recent years, no specific and targeted antifibrotic treatment strategies have been approved, potentially due to the complicated molecular mechanism that initiates and drives the fibrotic events, which remains to be completely understood. In this review, we aimed to discuss the epidemiology, etiology, and risk factors associated with the OSF, with special emphasis on the recent developments such as the use of flavored areca nut, *etc.* Then we highlight the OSF pathogenesis with special emphasis on the role of TGF-b, epithelial-mesenchymal transition, and other processes such as dysregulation of collagen metabolism and angiogenesis. We also mentioned the role of hypoxia-induced pathogenesis, which recently has been more in focus. Next, apart from traditional diagnosis methods, i.e., clinical evaluation and histopathology, we also discussed newer techniques such as biomarkers present in serum, saliva, and tissue biopsies. Afterwards, we mention ongoing traditional and modern treatments in clinical settings, such as the use of natural compounds, anti-fibrotic agents, targeted therapy, and more. We also discussed the role of emerging new therapeutic targets and how targeting them can overcome the current limitations. Moving ahead, we discussed how next-generation sequencing and artificial intelligence have improved our understanding of OSF pathophysiology. We conclude with a discussion of future perspectives and potential ways for developing novel OSF treatment or management.

## 1 Introduction

Oral submucous fibrosis (OSF) is a chronic disorder that frequently occurs in buccal mucosa that may become cancerous and is mostly linked to eating areca nut (AN). It is marked by growing submucosal fibrosis, which makes it challenging to open the mouth due to burning sensations (Shih et al., 2019). OSF pathological characteristics comprise chronic inflammation, excessive accumulation of collagen below the oral mucosal epithelium region, inflamed lamina propria and other connective tissues, and muscle degeneration. In addition to burning sensations, OSF patients also experience dry mouth, pain, restricted mobility of the tongue, taste disorders, dysphagia, trismus, and altered tone. The World Health Organization (WHO) has declared OSF one of the oral malignant disorders, and as per the report by Murthy et al., it has a transformation rate of ∼6% into oral squamous cell carcinoma (OSCC) (Murthy et al., 2022). OSF incidence varies with ethnicity and region and depends on diet, habits, and culture (Singh et al., 2021). As per a recent study by Wang et al., the overall prevalence rate of OSMF is about 3% (95% CI 2.8%–3.2%) worldwide and 4% (95% CI 3.7%–4.3%) in India (Wang et al., 2024a). Oral cancer makes up more than 30% of all cancer.

OSF is caused by several factors, such as autoimmunity, deficiency of iron, vitamin B, and C, betel nut chewing, spicy food consumption, human papilloma virus (HPV) infection, and genetics (Balakrishnan and Aswath, 2015; Wang et al., 2015). Among these, betel nut chewing is one of the most significant risk factors, which is further amplified if smoking and drinking are also involved. For example, a study showed that 86% of OSF patients in Taiwan are tobacco smokers and 74% of them are alcohol drinkers. Oral submucous fibrosis (OSF) is a chronic, progressive, and potentially malignant condition. The pathogenesis of the OSF is multifactorial, which includes dysregulated collagen metabolism, TGF-β activation and receptor binding, epithelial-mesenchymal transition (EMT), biphasic pattern angiogenesis, inflammation, and more.

**FIGURE 1:**
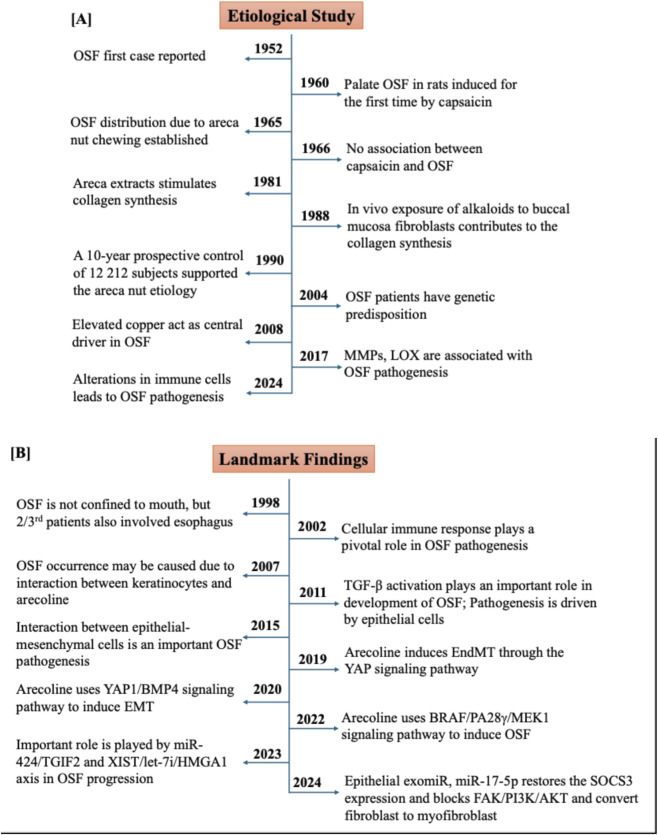
Key events in the oral submucosal fibrosis. **(A)** Describes the major etiological studies of OSF while **(B)** describes the major relevant studies of OSF.

OSF is widely recognized as a premalignant condition and precursor to oral precancer. As per previous reports, in China, 1.19% of OSF patients develop oral cancer; in India, ∼7.6% of patients develop oral cancer (Shih et al., 2019). Direct association exists between developing oral cancer and the duration and symptom level of OSF. Usually, OSF transitions into oral cancer in a time frame of 3–16 years after initial diagnosis (Shen et al., 2020). Despite various treatment approaches, including corticosteroids, antioxidants, and natural compounds, there are no effective treatments available in clinical settings. Advancements in the next-generation sequencing (NGS) technologies have allowed researchers to generate multi-omics data (transcriptome, epigenome, proteome, and metabolome) to characterize newer diagnostic and prognostic markers as well as new therapeutic targets. We have provided the key milestones in the development linked with OSF etiology and pathogenesis over the period of time (Figure 1).

## 2 Etiology and risk factors

OSF prevalence rate varies from ∼0.1% in China, 0.04%–6.4% in various parts of India (Wang et al., 2024b), to 0.15%–14.5% in Vietnam (Reichart and Nguyen, 2008). Of over 600 million people in the world who chew AN, nearly 5% suffer from OSF, establishing a link between OSF and AN chewing (Athukorala et al., 2021). Furthermore, the rate of transformation from OSF into oral cancer is above 4% worldwide; in India, the overall transformation rate is nearly 6% but varies within cities (9.8% in Lucknow, 2% in Mumbai). Epidemiological data further revealed higher OSF incidence among females in certain populations, suggesting possible hormonal or nutritional influences. Furthermore, the geographical and ethnic distribution of OSF incidence suggests the presence of underlying systemic or genetic predispositions. However, the etiology of OSF remains unknown and is widely believed to be multifactorial. Both local irritants and systemic factors are believed to play pivotal roles in the onset and development of OSF. We summarize these factors and their roles below.

### 2.1 Local causative factors

AN chewing (normal or flavored) is considered the most significant causative factor of OSF. The consumption rate in OSF patients ranges from 34% to as high as 100%. One such study showed that chewing AN alone causes OSF in 43% of the population, reporting chemical and physical irritation, as mentioned by the International Agency for Research on Cancer (IARC). This chemical irritation is caused by the AN constituents, which include alkaloids, flavonoids, tannins, carbohydrates, proteins, and fats, with arecoline and arecaidine (alkaloids) being the most irritating, causing abnormal collagen accumulation. Beyond AN, there are other factors linked with OSF pathogenesis. These include capsaicin (the active pungent component in chillies), consumption of pungent and spicy foods, alcohol, and pan (betel leaf often combined with tobacco powder and slaked lime). A few studies have also highlighted the role of bacterial, fungal, or viral infection in OSF; however, the exact mechanism remains unclear (Bandara and Samaranayake, 2019). Chronic mechanical irritation (CMI) is another factor linked with OSF pathogenesis, where pathological changes, including atrophy, ulceration, keratosis, hyperplasia, and even fibrosis, will appear in the oral mucosa under direct contact with a ‘mechanical agent’ such as a denture or teeth.

Studies have reported the direct evidence of autoimmune factors in OSF pathogenesis. For instance, Wang et al. demonstrated the presence of elevated incidence of anti-nuclear antibodies (ANA) in OSF patient sera. Using a human proteome microarray, the authors showed *PTMA* as a novel OSF-specific autoantigen, linked with fibroblast proliferation and ECM regulation in a TGF-β1-induced cell fibrosis model. Chiang et al. made a similar observation and reported increased levels of ANA, anti-smooth muscle (SMA), and anti-gastric parietal cell (GPCA) in OSF patients (Chiang et al., 2002). In summary, the findings indicate the role of immunoregulatory abnormalities in OSF pathogenesis.

### 2.2 Systemic factors

Similarly to local irritants, systemic factors also contribute to the OSF pathogenesis. These factors include dysregulation in vitamin, protein, and lipid levels; lack of proper, balanced, and nutritious diets; increased level of copper; and decreased level of zinc and iron (Tang et al., 2025). Jin et al. demonstrated iron deficiency as a cause rather than a disease consequence, possibly due to its role in epithelial atrophy and mucosal vulnerability. The patients also experienced burning mouth syndrome (BMS), lower blood hemoglobin (Hb) and serum iron, vitamin B12, and higher serum levels of homocysteine (Jin et al., 2020). The prolonged state of nutritional deficiency, such as vitamin B12, may condition the oral mucosa to be more susceptible to irritants, which could explain the higher prevalence of OSF in females, who may be more prone to such deficiencies. Just like iron, copper is another element associated with OSF, where its elevated level has been observed. It regulates one of the key enzymes in OSF pathogenesis, lysyl oxidase (LOX), a copper-dependent monoamine oxidase. A higher enzyme activity of LOX has been observed in fibroblast cells, where it promotes the cross-linking of collagen and its deposition.

Increased frequencies of genes such as human leukocyte antigen (HLA) (Purohit et al., 2025), cytotoxic T lymphocyte-associated antigen-4 (CTLA-4) (Shin et al., 2004), glutathione s-transferase (GST) (Motahari and Neshanifard, 2025), cytochrome P450 (CYP) (Li et al., 2011), and matrix metalloproteinase (MMPs) (Kazmi et al., 2022), as well as haplotypic combinations such as A10/DR3, B8/DR3, and A10/B8 have been observed in OSF patients. Additionally, studies have demonstrated cytogenetic damage in the form of sister chromatid exchanges (Jeyapradha et al., 2011), micronucleus cell numbers, and the degree of DNA damage (Paulose et al., 2016).

## 3 Pathogenesis and progression of oral submucous fibrosis (OSF)

**FIGURE 2:**
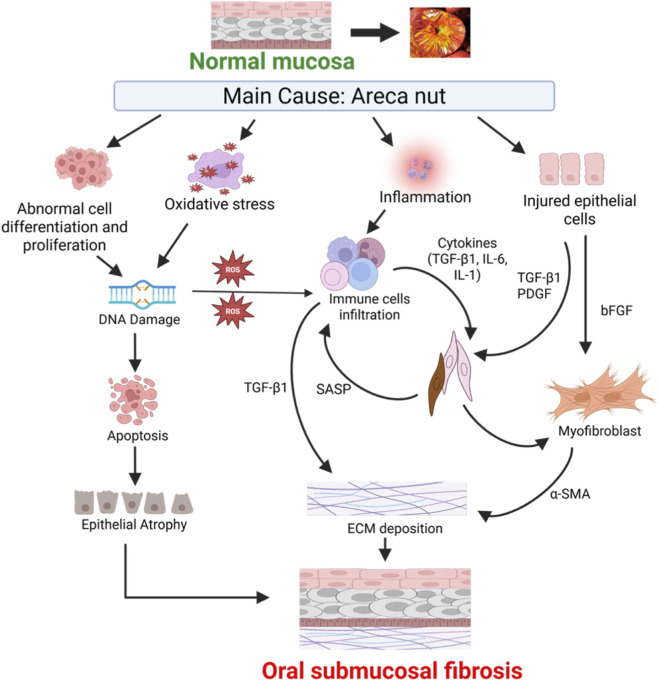
Pathogenesis of oral submucous fibrosis induced by Areca nut. Oral submucous fibrosis (OSF) is the result of pathological alterations in normal oral mucosa following chronic Areca nuts exposure. Areca nut trigger abnormal cell differentiation and proliferation, oxidative stress causing DNA damage leading to generation of reactive oxygen species (ROS) and epithelial atrophy. Areca nut also induces chronic inflammation and recruitment of immune cells, which secrete profibrotic cytokines that activate fibroblasts to produces excessive ECM. In addition, areca nut-induced epithelia cells stimulate the secretion of bFGF, which activates myofibroblasts and drives ECM deposition.

Oral submucous fibrosis (OSF) is a chronic, progressive, and potentially malignant condition and is considered an excessive repair process occurring post persistent chronic injury. In this context, all types of oral mucosal cells, including epithelial cells, fibroblasts, endothelial cells, and immune cells that have infiltrated the tissue, contribute to the development of OSF pathogenesis. Thus, a proper understanding of the molecular and cellular OSF mechanism is of paramount importance. The pathogenesis of the OSF is multifactorial and works in a complex interplay between host immune responses, exogenous compounds obtained from AN, epithelial-stromal crosstalk, and genetic predispositions (Figure 2). Histopathologically, OSF is marked by juxta-epithelial inflammatory reactions, increased fibrosis in the lamina propria and deeper connective tissues, epithelial atrophy, and, in a few cases, malignant transformation. Over the past few decades, our understanding of the biological basis of OSF has changed a lot, especially with growing research into molecular fibrogenesis, epithelial-mesenchymal transition (EMT), and the tumor microenvironment.

### 3.1 AN constituent

Habitual betel nut chewing plays a pivotal role in OSF initiation by means of a multitude of compounds (alkaloids, polyphenols, and trace metals) released into the oral cavity. Alkaloids, primarily arecoline, are the key mediators of fibrogenic activity, leading to persistent and chronic injury followed by massive fibroblast activation. When proliferating, fibroblasts produce a matrix with a phenotype of skeleton proteins such as α-SMA and ECM components including tenascin, collagen type I, and fibronectin (Younesi et al., 2024), establishing a significant correlation with the disease severity in OSF patients (Angadi et al., 2011). In addition, elevated levels of reactive oxygen species (ROS) and nitric oxide (NO) have been observed when fibroblasts are exposed to arecoline in a time- and dose-dependent manner, demonstrating the role of oxidative stress. At the same time, tannins and flavonoids are associated with the collagen cross-linking, making the fibers more resistant to enzymatic degradation. This results in the gradual buildup of insoluble collagen in the submucosa, which is a key feature of OSF.

### 3.2 TGF-β activation and TGF-β receptor binding

TGF-β, a key fibrogenic cytokine, is another factor that is associated with OSF pathogenesis. Its production is stimulated by AN and its constituents and acts as a master regulator of fibrotic events *via* activating numerous downstream pathways linked with OSF progression (Meng et al., 2016). TGF-β comprises three isoforms, i.e., β1, β2, and β3. Fibrogenic activity is mediated by all these isoforms, with β1 identified as the most potent isoform. TGF-β, upon activation, binds to TGF-β receptors, types I and II. These receptors belong to the serine/threonine kinase family and are expressed on epithelial and fibroblast cells. The ligand-receptor interaction of TGF-β and its receptor accounts for several key molecular and cellular functions, including activation of the canonical Smad signaling pathway (Smad2 and Smad3); profibrotic gene expression regulation (Pant et al., 2016); fibroblast proliferation; increased collagen synthesis; and phenotypic conversion of fibroblasts to myofibroblasts (MFBs). Other than canonical pathways, TGF-β also accounts for fibroblast activation *via* inducing non-canonical pathways such as JNK and p38 MAPK (Chang et al., 2013; Pant et al., 2016). It not only synthesizes collagen but also suppresses the activity of enzymes associated with collagen degradation, such as matrix metalloproteinases (MMPs), by upregulating their inhibitors, TIMPs (tissue inhibitors of metalloproteinases). TGF-β also accounts for phenotypic changes of fibroblasts into α-smooth muscle actin (α-SMA)-positive MFBs (contractile cells regulating wound contraction and fibrosis) (Angadi et al., 2011).

### 3.3 Angiogenesis biphasic pattern

The biphasic pattern of angiogenesis is another crucial hallmark of OSF. In the first phase, clinicians reported significant reduction in the blood vessels in the early or moderate stage. Studies have shown the synthesis and accumulation of excessive collagen around the blood vessels, making them thinner and occluded. This condition leads to the tissue ischemia and creation of a hypoxic environment (Agarwal et al., 2022), which in turn triggers the hypoxia-inducible factor-1a (HIF-1α) stabilization, leading to enhanced TGF-β transcription and perpetuated fibrogenesis (Mingyuan et al., 2018). In the second phase, the pattern reverses during oncogenic transformation. Here, expression of vascular endothelial growth factor (VEGF) elevates along with the formation of new blood vessels, suggesting an adaptive response toward the nutritional requirement of dysplastic and potentially neoplastic tissues.

### 3.4 Epithelial-mesenchymal transition

EMT is another molecular axis that has gained prominence in recent OSF research as epithelial cells maintain cell wall integrity and homeostasis in oral submucosa. It is defined as a phenomenon where epithelial cells lose their polarity and cell-cell adhesion property, adopting characteristics of mesenchymal cells such as motility and invasiveness. Studies have reported how an AN extract causes damage to the epithelial cells and exhibits a significant toxic effect on keratinocyte proliferation and induces cell death in a dose-dependent manner (Jeng et al., 2003). In this process, chronic inflammation and TGF-β signaling create a pro-EMT microenvironment, which is apparent by the loss of epithelial cell markers (E-cadherin) and gain of mesenchymal cell markers (α-SMA, vimentin, N-cadherins, *etc.*). These changes in epithelial cells have been shown to promote the OSF by maintaining the activation of some key profibrotic pathways (Shetty et al., 2020). For instance, Xie et al. showed that arecoline upregulates levels of proteins including PA28γ, BRAF, and phosphorylated MEK1. These proteins interact with each other to form a complex, which in turn activates the MEK1/ERK signaling pathway and promotes EMT in oral epithelial cells (Xie et al., 2022). Beyond pathogenesis, EMT hints at a mechanistic link between OSF and OSCC, where an AN extract (alkaloids and flavonoids) can lead to chronic injury and inflammation, leading to tissue remodeling and malignant phenotypes (Shetty et al., 2025). Furthermore, recently, immunohistochemistry and RNA-sequencing studies have confirmed that EMT markers are upregulated in OSF tissues, particularly in regions showing dysplasia or carcinoma *in situ* (Hosur et al., 2021).

### 3.5 Dysregulated collagen metabolism

In parallel to various signaling cascades, dysregulation in collagen metabolism and homeostasis also contributes to OSF’s pathogenesis (Amar et al., 2017). Major causes of this dysregulation include exposure to AN constituents, alcohol drinking, and tobacco smoking, resulting in overexpression of procollagen mRNA and ultimately leading to excessive production of collagen types I and III (Ala-Kokko et al., 1987). Another factor linked with OSF pathogenesis is reduced collagen clearance, which takes place due to several reasons, including increased collagen stabilization, dysregulated ECM dynamics, and phagocytosis inhibition (Arakeri et al., 2017). The key reason for the non-degradable and tightened collagen matrix is because of the upregulation of Tissue Inhibitor of Metalloproteinases (TIMPs), which suppresses MMP activity (Cabral-Pacheco et al., 2020), and LOX activity, which is responsible for the intra- and inter-molecular cross-linking of non-degradable (proteolytic degradation) collagen fibrils (Cai et al., 2017).

OSF histological progression can be categorized into four stages based on collagen formation and accumulation, which include (i) very early, (ii) early, (iii) moderately advanced, and (iv) advanced. The first stage is characterized by loose collagen bundles and mucosal edema. The second stage is marked by the hyalinization onset and vascularity reduction. In the third stage, we see more pronounced fibrosis with densely packed collagen and obliterated vessels, and lastly, in the last stage, there is more pronounced fibrosis characterized by densely packed collagen and obliterated vessels. Lastly, in the advanced stage, collagen becomes heavily cross-linked, and phenotypic changes of fibroblasts to myofibroblasts (MFBs) along with an atrophic epithelium are observed, which increases the risk for epithelial dysplasia and oral squamous cell carcinoma (OSCC) (Passi et al., 2017).

### 3.6 Inflammation

Inflammation is a key driver in OSF pathogenesis, both as a cause and consequence of fibrotic change. AN chewing triggers a persistent inflammatory reaction marked by the immune cells’ infiltration, such as lymphocytes, macrophages, plasma cells, and occasionally eosinophils (Zhang et al., 2023). These immune cells release a cascade of pro-inflammatory cytokines, including interleukin-1 beta (IL-1β), interleukin-6 (IL-6), tumor necrosis factor-alpha (TNF-α), and interferon-gamma (IFN-γ). Such cytokines increase the inflammatory response, influence the fibroblast behavior, stimulate their proliferation, and secrete ECM components. This fibrotic cascade is further fueled by the crosstalk between immune cells and stromal fibroblasts, facilitating a microenvironment for neoplastic transformation (Zhang et al., 2024). Interestingly, as the stage progresses, the increased mast cell population starts to decline (Pujari and Vidya, 2013). This pattern of immune cell infiltration helps in delineating histopathological staging as well as reflects shifts in the local immune response during disease progression.

### 3.7 Effects on oral mucosal tissue

OSF symptoms develop worse as it progresses, and one primary and early complaint is a burning sensation in the oral mucosa while consuming spicy food. With the disease progression, patients experience reduced mouth opening (trismus), difficulty in eating, talking, and maintaining oral hygiene (Speight et al., 2018). Involvement of the soft palate, pharynx, and masticatory muscles further exacerbates functional limitations (Shankar et al., 2014). Depending on the population investigated and the severity of the disease, the probability of OSF turning into cancer ranges from 7% to 13% (You et al., 2023). Dysplastic alterations, leukoplakia, persistent ulceration, and high epithelial mitotic indices are all signs of potential malignant evolution (Kumari et al., 2022).

## 4 Oral submucosal fibrosis diagnosis

### 4.1 Differential classification

OSF classification has been well documented in multiple forms, including clinical, functional, and histological. Clinicians use certain staging systems for OSF clinical diagnosis and treatment (Passi et al., 2017). As per the clinical staging system, OSF patients with stomatitis and vesiculation are classified into the early stage; patients with marble-like appearance and palpable fibrous bands into the moderate stage; and patients with leukoplakia and erythroplakia are classified into the severe stage. As per functional classification, the patient is classified into the I-V range based on maximum interincisal mouth opening from >35 mm (Stage I) to <5 mm (Stage V). As OSF transforms into OSCC, solid biopsies from patients are essential to assist with clinical diagnoses and designing therapeutic strategies. In addition, biomarkers such as mRNAs, non-coding RNAs, and proteins are also implicated in OSF staging and classification. Recently, liquid biopsies from sera and saliva have been used to extend the measuring instrument’s functionality.

### 4.2 Tissue biopsy

Tissue staining is the most common and widely used technique for obtaining histological images from solid biopsies. Experimental techniques such as PCR (methylated, real-time), western blotting, and staining are employed to detect biomarkers. It allows us to identify promoter methylation, gene and protein expression levels, and marker location in the tissues.

#### 4.2.1 Stain based

H&E staining is used as a control for immunohistochemical (IHC) staining, as it demonstrates that tissue has been processed appropriately and reveals artifacts. The technique stains the nuclei purple and the cytoplasm pink, elucidating basic tissue morphology as well as facilitating pathologists diagnosing OSF. Cellular and molecular changes such as alterations in epithelial cells, rete-peg shapes, subepithelial depositions of collagen fibers, dense bands, and inflammatory cells serve as OSF markers. Comparative assessment of three different strains, i.e., Mallory’s, Masson’s, and Van-Gieson performance with H&E staining on 30 OSF tissues, demonstrated the superiority of Mallory’s stain, as it showed variations in the thick keratin layer of several tissues, including stratified squamous epithelium, degenerating skeletal muscle bundles, *etc.* However, the stain failed to demonstrate any constricted blood vessels (Reshma et al., 2016).

#### 4.2.2 Coding genes and proteins as diagnostic biomarkers

The pathology of OSF implicates several molecules and pathways, including hypoxia and EMT. Most cases have shown positive expression of proliferating cell nuclear antigen (PCNA) in basal, suprabasal, and superficial layers (Keshav and Narayanappa, 2015). Proteomic two-dimensional electrophoresis (2-DE) characterizes cyclophilin A (CYPA) as a biomarker and target of OSF (Hou et al., 2017), where it promotes carcinogenesis by increasing cell division and preventing apoptosis *via* caspase deactivation (Yuan et al., 2016). Likewise, matrix-assisted laser desorption imaging mass spectrometry (MALDI-IMS) characterized nuclear receptor coactivator 7 (NCOA7) as another potential OSF biomarker, which was further confirmed in cell lines, animal models, and 32 paired OSCC and adjacent normal tissues. Proteins linked with NCOA7 regulate cell cycle and cell division, marking them as potential biomarkers for the early OSF malignant transformation diagnosis (Xie et al., 2016).

Arecoline induces HIF-1α protein in a concentration-dependent manner and is significantly upregulated in fibroblasts, epithelial cells, and inflammatory cells of nut chewers. This activated HIF-1α further stimulates the PA-1 expression, induces ECM accumulation, and leads to OSF (Tsai et al., 2015). CD105, a TGF-β signaling receptor, is linked with fibrogenesis, angiogenesis, and endothelial cell proliferation and serves as a more specific biomarker than CD34 when implied in determining OSF neo-angiogenesis (Pammar et al., 2018). Similarly, the combined approach of 1D SDS-PAGE (one-dimensional sodium dodecyl sulfate polyacrylamide gel electrophoresis) and nanoLC (nanoliquid chromatography) observed a higher expression of α-enolase in the tissue of OSF patients with dysplasia compared to normal oral mucosa, marking it as another potential biomarker. The enzyme regulates the PI3K/AKT signaling pathway by promoting cell division, activates plasminogen and induces tumorigenesis, and increases the Warburg effect. Overexpression of α-enolase can be detected by various techniques such as western blotting, IHC, and RT-qPCR (Bag et al., 2018). Beyond the aforementioned biomarkers, additional biomarkers have been observed, including Ki67 and cyclin D1, which determine cell proliferation; p16 and p53, which cause tumor suppression; hepatocyte growth factor receptor c-Met; and the insulin-like growth factor II mRNA-binding protein 3 (IMP3), which is associated with tumor invasion (Bazarsad et al., 2017).

#### 4.2.3 Non-coding genes as diagnostic biomarkers

Several non-coding genes have also been reported as potential biomarkers for OSF diagnosis. For example, microRNAs miR-200b and miR-200c were found to be downregulated in OSF. The miRNAs miR-200b and miR-200c target ZEB1 and ZEB2, respectively, leading to E-cadherin upregulation (Liao et al., 2018; Lu et al., 2018). ZEB1 binds to the promoter region of α-smooth muscle actin (α-SMA), upregulating it in the myofibroblasts during fibrogenesis. Another such marker is LncRNA GAS5-AS1, which is downregulated in OSF tissue. It represses phosphorylated Smad2 and downregulates the expression of TGF-β/Smad and α-SMA in myofibroblast cells (Lin et al., 2018). In contrast, opposite behavior for LINC00974 has been observed, where it is aberrantly upregulated in myofibroblasts and OSF tissues (Fang et al., 2019). Table 1 provides the summary of the tissue biomarkers as given below:

**TABLE 1:** Tissue based biomarkers in OSF diagnosis. The table summarizes the list of the reported biomarkers characterized from the tissue biopsy, its gene expression status, pathways or processes involved and the sample size of the study.

Biomarker	Expression	Pathway/Process	Sample size	References
PCNA	Up	Associated with cell proliferation	30 OSF, 10 OSCC	Keshav and Narayanappa (2015)
Cyclophilin A	Up	Associated with cell proliferation	25 control, 25 OSF	Yuan et al. (2016), Hou et al. (2017)
NCOA7	Up	Role in cell proliferation; associated with early diagnosis of OSF malignant transformation	24 OSF tissues without malignant transformation, 34 OSCC tissues arising from OSF	Xie et al. (2016)
HIF-1α, PAI-1	Up	Associated with angiogenesis	6 control, 25 OSF	Tsai et al. (2015)
CD105	Up	Associated with angiogenesis	15 control, 30 OSF	Pammar et al. (2018)
α-enolase	Up	Associated with cell proliferation and tumorigenesis	13 control, 19 OSF without dysplasia (OSFWT), 23 OSF with dysplasia (OSFWD), 28 OSCC	Bag et al. (2018)
Ki67, cyclin D1, c-Met, IMP3	Up	Ki67, cyclin D1 are associated with cell proliferation, c-Met, IMP3 play role in invasion	6 control, 36 OSF	Bazarsad et al. (2017)
β-catenin	Down	Associated with cell proliferation	6 control, 36 OSF	Bazarsad et al. (2017)
WIF1	Down	Act as Wnt antagonist, binds to Wnt protein and inhibits Wnt/β-catenin signalling by directly	15 control tissue, 45 OSF, 55 OSCC	Zhou et al. (2015a)
β-catenin	Up	Play a crucial role in Wnt/β-catenin signalling	15 control, 45 OSF, 55 OSCC	Zhou et al. (2015b)
SFRP-1, SFRP-5	Down	Play a crucial role in Wnt/β-catenin signalling	15 control, 45 OSF, 55 OSCC	Zhou et al. (2015b)
miR-200b, miR-200c	Down	ZEB2 is targeted by miR-200b, ZEB1 is targeted by miR-200c	25 normal, 25 OSF; 20 normal, 20 OSF	Liao et al. (2018), Lu et al. (2018)
ZEB1, ZEB2	Up	ZEB2 is targeted by miR-200b, ZEB1 is targeted by miR-200c	25 normal, 25 OSF; 20 normal, 20 OSF	Liao et al. (2018), Lu et al. (2018)
LncRNA GAS5-AS1	Down	LncRNA GAS5-AS1 bind to Smads and prevents them binding to SBE on TGF-β target gene promoter, thereby negatively regulates TGF-β/Smad signaling pathway	25 normal, 25 OSF	Lin et al. (2018)
LncRNA LINC00974	Up	LncRNA LINC00974 activates TGF-β/Smad signaling	20 OSF tissues	Fang et al. (2019)

### 4.3 Liquid biopsy

Modern detection techniques are achieving increasing stability and sensitivity and can detect increasingly smaller amounts of free ions, circulating cells, enzymes, nucleic acids, and proteins in body fluids such as saliva, serum, *etc.* (More et al., 2017). Compared to normal tissues, OSF tissues showed elevated serum proteins and globulin levels. Likewise, when OSF transforms into OSCC, higher levels of serum copper have been observed along with the betel quid chewing duration (Hosthor et al., 2014). Recently, biomarkers specific to OSF have been identified from serum and saliva (Table 2). They have been used for diagnosis, which can be further improved with the increase in sample size. These are discussed in detail below.

**TABLE 2:** Liquid biopsy-based biomarkers in OSF diagnosis. The table summarizes the list of the reported biomarkers characterized from the liquid biopsy, its gene expression status, pathways or processes involved and the sample size of the study.

Biomarker	Site	Expression	Pathway/Process	Sample size	References
Serum protein, globulin	Serum	Down	-	50 control, 50 nicotina stomatitis, 50 OSF, 50 leukoplakia, 50 malignancy	More et al. (2017)
Copper	Serum	Up	Serum copper levels increased gradually from precancer to cancer, as the duration of betel quid chewing habit increased	30 control, 30 OSF, 30 OSCC	Hosthor et al. (2014)
Sister chromatid exchange in lymphocytes	Serum	Up	Genotoxic, genome instability	10 male patients who had the habit of chewing pan for 5 or more years, 10 male patients with OSF who had panparag chewing habit and 10 age- and sex-matched controls without any chewing habit	Jeyapradha et al. (2011)
β-carotene	Serum	Down	β-carotene as the best-known provitamin A carotenoid	40 control, 40 OSF	Aggarwal et al. (2011)
E-SOD, GPx	Serum	Down	Anti-ROS stress	25 control, 25 OSF, 25 leucoplakia, 25 OSCC	Gurudath et al. (2012)
LDH	Serum	Up	Cell metabolism	30 control, 30 OSF; 20 control, 20 OSF	Mishra et al. (2018)
MDA, comet assay	Serum	Up	ROS product (MDA), DNA damage phenotype (comet assay)	30 control, 30 OSF	Paulose et al. (2016)
8-OHdG	Saliva	Up	Produced by ROS, reduces anti-ROS stress	40 OSF, 40 control, 40 OSCC, 40 oral lichen planus lesions	Kaur et al. (2016)
MDA	Saliva	Down	Produced by ROS, reduces anti-ROS stress	40 OSF, 40 control, 40 OSCC, 40 oral lichen planus lesions	Kaur et al. (2016)
GPx	Saliva	Down	Reduces anti-ROS stress	63 normal and 63 OSF	Divyambika et al. (2018)
SOD	Saliva	Down	Reduces anti-ROS stress	63 normal and 63 OSF	Divyambika et al. (2018)
LDH	Saliva	Up	It catalyzes oxidation of lactate to pyruvate	20 normal and 20 OSF	Mishra et al. (2018)
S100A7	Saliva	Up	Calcium binding small protein; causes psoriasis and carcinoma in several epithelial types	30 normal and 30 OSF	Raffat et al. (2019)
bFGF	Saliva	Up	Multiple pathways (MAPK, PI3K, and mTOR)	90 control and case	Shafaq Saeed Roghay et al. (2025)

#### 4.3.1 Serum-based biomarkers in OSF

Compared to healthy controls, the rate of sister chromatid exchange per lymphocyte is higher in OSF patients. This genome instability is caused by ROS-induced DNA damage (Jeyapradha et al., 2011). Likewise, provitamin A carotenoid β-carotene is another biomarker in OSF, whose levels decreased with the disease progression (Aggarwal et al., 2011); levels of enzymes such as erythrocyte superoxide dismutase (E-SOD) and glutathione peroxidase (GPx) were significantly lower in the OSF, oral leukoplakia, and oral cancer groups than the control (Gurudath et al., 2012). Elevated levels of lactate dehydrogenase (LDH) have been observed in oral cancer and malignant lesions/conditions. A direct correlation between serum LDH level and mouth opening was observed; however, no such correlation was observed with salivary LDH (Mishra et al., 2018), suggesting that serum LDH may be a better biological marker of OSF. Higher levels of peroxidation have been observed in OSF compared to the control. One such lipid peroxidation marker is malondialdehyde (MDA), whose level estimate using comet assay can help identify OSF patients with high malignant potential (Paulose et al., 2016).

#### 4.3.2 Saliva-based biomarkers in OSF

Several biomarkers have been identified in the saliva of OSF patients, such as MDA, 8-hydroxy-2-deoxyguanosine (8-OHdG), vitamin C, and vitamin E. These markers were found to be higher in OSF patients compared to normal controls. However, lower concentrations of vitamins C and E were present in OSF compared to the control. These multiple biomarkers could increase sensitivity and specificity in OSF diagnosis (Kaur et al., 2016). Likewise, in a separate study, total salivary protein and lipid peroxides were found to be higher; however, salivary SOD, GPx, and vitamins A, C, and E were lower in OSF patients compared to the control (Divyambika et al., 2018). Salivary S100A7 is another such marker whose high expression has been observed in malignant oral disorders and is related to the malignant transformation risk in oral dysplasia (Zhou et al., 2008; Raffat et al., 2019). Recently, Roghay et al. reported basic fibroblast growth factor (bFGF) as another promising non-invasive salivary biomarker for early detection of OSF (Shafaq Saeed Roghay et al., 2025).

bFGF has been reported as a multi-functional growth factor and plays an important role in key processes such as inflammation, wound healing, angiogenesis, tissue remodeling, and more, all of which are associated with OSF pathogenesis (Zare et al., 2023). In the case of OSF, bFGF has been reported to be secreted by activated epithelial cells, fibroblasts, and inflammatory cells, leading to higher fibroblast proliferation, myofibroblast differentiation, and ECM deposition. bFGF also promotes pathological angiogenesis and epithelial-mesenchymal interactions, thereby creating a microenvironment that is profibrotic in nature. Increased levels of bFGF in saliva in OSF patients mostly highlight these localized mucosal and stromal events, making saliva a biologically relevant surrogate for disease activity (Gorugantula et al., 2012). In contrast, cases reporting bFGF from OSF patients’ serum are very limited, potentially because of dilution effects and other systemic inflammatory conditions (Bishen et al., 2008). These findings established the salivary bFGF as an active fibrotic and inflammatory signal in OSF, highlighting its biological and clinical relevance.

### 4.4 Instrument usage for OSF diagnosis

Several modern and advanced instruments are routinely used for clinical diagnosis of OSF. Tissue biopsies are used for clinical diagnosis; however, certain patients refuse incisional biopsies. In these cases, techniques such as autofluorescence spectroscopy, optical coherence tomography (OCT), and Fourier transform infrared spectroscopy (FTIR) come into the picture, where they assist incisional biopsy in the diagnosis of OSF. Autofluorescence spectroscopy takes advantage of the fact that diseased tissues have diverse and unique histomorphological features. When tissues are activated at the right wavelength, intrinsic fluorophores rise to different fluorescence emission spectra. For example, maximum emission for tryptophan is measured at 340 nm, for collagen in between 380 and 440 nm, and for nicotinamide adenine dinucleotide (NADH) in between 440 and 460 nm. In the case of OSF, the 330 nm excitation application leads to a significantly higher peak of 380 nm and a lower peak of 460 nm than those of normal oral mucosa (Chen et al., 2003). A significant difference in emission peak was further identified between normal and OSF patients and between OSF patients and betel quid chewers (Ponnam et al., 2012). Post-treatment, the mucosa exhibited relatively lower intensity at ∼385 nm and comparatively higher intensity at ∼440 nm than untreated OSF mucosa (Vedeswari et al., 2009). In 1988, for the first time, the OCT system was used on human teeth and oral mucosa. The instrument uses low-coherence light to capture 2D and 3D images at micrometer resolution. The thickness of the epithelium and the standard deviation (SD) of the A-mode scan intensity in the lamina propria layer are effective diagnostic markers of OSF (Lee et al., 2009).

### 4.5 Combination of serum and instrumentation in OSF diagnosis

FTIR generates infrared absorption and emission spectra for states of matter, i.e., solids, liquids, and gases. Furthermore, as mentioned above, the level of protein, globulins, lipids, vitamins, copper, enzymes, nucleic acids, *etc.*, Present in the serum varies significantly in OSF compared to the control. Combining the two strategies, i.e., instrumentation and serum, is more effective than either approach alone and further saves time and reagents on OSG diagnosis. Hence, FTIR spectroscopy of OSF patients’ sera could be useful for preoperative screening or diagnosing more effectively and accurately (Rai et al., 2018). Currently, more solid-biopsy-based biomarkers are present than liquid-biopsy-based ones.

## 5 Treatment strategies for OSF

**FIGURE 3:**
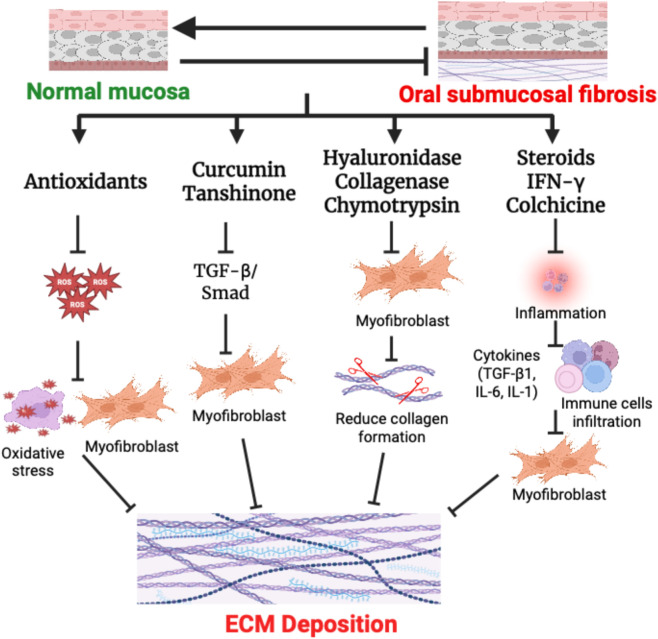
Treatment strategies for OSF regression: Treatment with antioxidants supress ROS generation and thereby reduce oxidative stress and ECM deposition. Natural compounds such as curcumin and tanshinone act on TGF-β/Smad signaling pathway and limiting ECM deposition. Local injection of hyaluronidase, collagenase and chymotrypsin reduce the levels of ECM deposition by hydrolysis of hyaluronic acid, esters and peptide bonds and reduction of collagen formation. In addition, immunomodulators such as steroids or anti-fibrotic cytokines inhibit inflammation and prevention of activation of immune cells and attenuating fibrosis.

The complexity of pathogenesis is responsible for the lack of effective therapeutic modalities against OSF. The primary purpose of the treatment is to relieve the patient’s symptoms to the maximum extent and prevent further disease progression. The current knowledge of the present treatment strategies is based on etiology, risk factors, pathogenesis, disease progression, and patient symptoms. The current treatment can be broadly categorized into three groups, i.e., (a) medical treatment, (b) physical exercise, and (c) surgery. Among these, medical treatment is given preferably, whereas physical exercise and surgery are recommended in severe cases. Therefore, there is a pressing need to characterize new drug targets and drugs against them. We have briefly discussed some emerging drug targets and potential drugs below, which may be considered for clinical application (Figure 3).

### 5.1 Emerging targets

Recent advancements in molecular research have illuminated new therapeutic targets and innovative treatment options designed to impede disease development and reverse fibrotic alterations. Below, we discuss emerging targets with high therapeutic potential.

#### 5.1.1 Transforming Growth Factor-beta

Transforming Growth Factor-beta 1 (TGF-β1) is well-known for driving fibrosis in OSF. It is essential for stimulating fibroblasts, increasing collagen synthesis, and promoting EMT. Currently, inhibiting TGF-β1 signaling has been a major therapeutic avenue, and intralesional corticosteroids are often used to do this. Alongside pharmacological inhibitors, natural substances like EGCG, a polyphenol present in green tea, have shown encouraging outcomes in both *in vitro* and *in vivo* models by markedly decreasing TGF-β1 expression and collagen accumulation (Gao et al., 2025).

#### 5.1.2 Lysyl oxidase (LOX)

Lysyl oxidase (LOX) is another choice of therapeutic target that is gaining more attention. LOX helps collagen and elastin fibers cross-link in the ECM, making the oral tissues stiffer in OSF, forming fibrotic bands harder to break down. Targeting this enzyme is a novel therapeutic strategy aimed at making the ECM softer and tissues more flexible. Polyphenols like EGCG and quercetin may help restore oral flexibility and lower the severity of fibrosis by lowering the expression and activity of LOX (Mehta et al., 2023).

#### 5.1.3 Reactive Oxygen Species (ROS)

Reactive Oxygen Species (ROS) also play a pivotal role in the OSF pathogenesis. AN exposure elevates the intracellular ROS levels, leading to oxidative stress, chronic inflammation, and fibroblast activation. Targeting ROS-mediated signaling and restoring redox homeostasis are key strategies under investigation. Researchers are looking into natural antioxidants like resveratrol and lycopene to see if they can inhibit ROS, protect against cellular damage, and inhibit fibrogenic signaling pathways (Fakhri et al., 2025).

#### 5.1.4 Cytokine-based therapy

Chronic inflammation is another hallmark of OSF, mediated by increased levels of pro-inflammatory cytokines such as IL-6, IL-8, and TNF-α (Haque et al., 1998). These cytokines help sustain an inflammatory microenvironment and facilitate fibroblast proliferation and collagen accumulation. As a result, targeting inflammatory signaling with molecules from the immunomodulator, anti-inflammatory drug, or phytochemical classes has opened new therapeutic avenues. Natural substances such as curcumin and quercetin possess strong anti-inflammatory properties and have shown efficacy in downregulating these cytokines in preclinical studies.

#### 5.1.5 Matrix metalloproteinases (MMPs)

In addition to these pathways, the abnormal remodeling of the extracellular matrix in OSF is largely caused by the dysregulation of matrix metalloproteinases (MMPs) and their natural inhibitors, tissue inhibitors of metalloproteinases (TIMPs). When TIMP activity is too high and MMP expression is too low, collagen builds up and the ECM turnover slows down. To improve matrix degradation and tissue remodeling, researchers are looking into ways to restore the MMP/TIMP equilibrium (Shrestha and Carnelio, 2013). EGCG and resveratrol are two examples of agents that can change the levels of MMP and TIMP, suggesting that they can help with both inflammation and fibrosis.

### 5.2 Current treatment

The current treatment for OSF includes surgery and noninvasive methods, including molecular approaches. Below, we discuss therapies for treating OSF that involve physical therapies, drug therapies, and compounds derived from natural extracts.

#### 5.2.1 Hyperbaric oxygen as a physical form of therapy (HBOT)

HBOT is typically used to treat diseases such as decompression sickness, gas gangrene, and carbon monoxide poisoning. In this procedure, the patient is placed in a special kind of hyperbaric chamber with high oxygen pressure. HBOT was applied for the first time in dentistry in 1988 to promote wound healing in the periodontal region. Recently, the HBOT application in the case of OSF has been reported, where it enhances apoptosis of fibroblasts and inhibits their activity by decreasing IL-1b and TNF-a production (Romero-Valdovinos et al., 2011; Oscarsson et al., 2017). HBOT lowers the levels of proinflammatory cytokines such as IL-1, IL-6, and IL-10 (Weisz et al., 1997); enriches oxygenation of all tissues; and prevents the production of ROS, for example, E-SOD, GPx, paraoxonase, catalase, and heme-oxygenase-1 (Cuzzocrea et al., 2000; Özden et al., 2004). In summary, HBOT suppresses fibroblast activity and exhibits anti-inflammatory and antioxidant properties, thus leading to the therapeutic effect of OSF (Ye et al., 2014).

#### 5.2.2 Drug treatment

The primary objective of the drug therapy in OSF is to reduce inflammation and ECM degradation. Steroid hormones belonging to the corticosteroid class are produced in the adrenal cortex of vertebrates and are primarily used to treat OSF. Two classes of hormones, i.e., glucocorticoids and mineralocorticoids, participate in several physiological and biochemical processes. Glucocorticoids majorly block inflammation mediators and slow down the inflammatory reaction (Liu et al., 2013). They also inhibit fibroblast proliferation and collagen deposition (Tsai et al., 1999).

Some of the synthetic drugs with glucocorticoid-like effects include dexamethasone, betamethasone, and methylprednisolone. Intralesional injection of these drugs has been found to significantly improve mouth opening (Tilakaratne et al., 2016; Sadaksharam and Mahalingam, 2017) and ease the burning sensation (Shetty et al., 2013) in OSF. Likewise, pentoxifylline, a xanthine derivative, improves mouth opening and alleviates the burning sensation during treatment of OSF patients and facilitates swallowing and speech (Marques et al., 1999). Another commonly used drug to treat OSF is colchicine, which was initially used to treat joint swelling since 1500 BC. The first report of colchicine for treating OSF was documented in 2013 (Krishnamoorthy and Khan, 2013). Colchicine is extracted from the autumn crocus plant. It reduces inflammation by preventing activation of neutrophils and their migration to the inflammation site and by suppressing IL-1β activation (Dalbeth et al., 2014). OSF patients are given 0.5 mg oral colchicine twice a day and receive an injection of 1500 IU hyaluronidase into each buccal mucosal lesion once a week. As a result, by the second week, mouth opening increases, and burning sensation and histological parameters reduce.

#### 5.2.3 Therapy using natural compounds

Natural compounds are extracted directly from living organisms with no additional modification. Most of these compounds, used to treat diseases, are extracted from herbs utilized in traditional chinese medicine (TCM) and the food we eat.

Some of the potential TCM compounds with efficacy against OSF include glabridin, butylidenephthalide, asiatic acid, tanshinone, and salvianolic acid B. Glabridin, a class of isoflavonoids, is extracted from the root of *Glycyrrhiza glabra* (licorice). It is a natural phenolic compound with antioxidant and anti-inflammatory properties and suppresses α-SMA, type I collagen, and TGF-β (Lee et al., 2018). Butylidenephthalide is another compound found to be effective against OSF. It is extracted from *Angelica sinensis* or *Ligusticum chuanxiong* and has several effects, including neuroprotective (Rajamani et al., 2017), anticancer (Chen et al., 2008), and vasorelaxant (Chan et al., 2006), and it inhibits inflammation and liver fibrosis (Chuang et al., 2016). The compound downregulates α-SMA, type I collagen, fibronectin, and myofibroblast bioactivity (Su et al., 2018). Asiatic acid, a type of TCM, has been found to be effective against OSF. It is extracted from *Centella asiatica* and ameliorates fibrosis of the lung (Dong et al., 2017) and liver (Fan et al., 2018). The compound suppresses TGF-β1, collagen 1 type 2, and collagen 3 type 1 in human buccal fibroblasts (Adtani et al., 2017). Likewise, tanshinone, another natural compound that is effective against OSF, is extracted from *Salvia miltiorrhiza.* This compound is a mixture of dihydrotanshinone I, tanshinone I, and tanshinone IIA and has activity such as antioxidant and anti-inflammatory. Tanshinone interacts with the p53 pathway, which is downregulated in OSF (Zheng et al., 2018). Salvianolic acid B, in a recent clinical trial, along with corticosteroids, has been shown to reduce burning sensation and improve mouth opening in OSF patients (Jian et al., 2017).

Additional natural compounds with potential anti-OSF efficacy include epigallocatechin-3-gallate (EGCG), aloe vera, lycopene, curcumin, and honey. EGCG, a catechin, is most abundant in tea. It is an antioxidant, and it exhibits its effect by suppressing cellular ROS (Hsieh et al., 2018). It has been shown that EGCG suppresses multiple fibrogenic genes such as early growth response-1, transglutaminase-2 (TGM-2), and connective tissue growth factor (Hsieh et al., 2015; Sar et al., 2015). Aloe vera, one of the widely used compounds with several therapeutic benefits, belongs to the family Liliaceae. Aloe vera contains several minerals and vitamins and possesses anti-inflammatory activity. It also lowers the inflammasome formation in human macrophages (Budai et al., 2013). Recent studies have observed the application of aloe vera in dentistry. It was found that it reduces the burning sensation of OSF within 2 months of treatment (Al-Maweri et al., 2019). Curcumin is a natural phenolic compound obtained from the *Curcuma longa* rhizomes. It is a common food additive and dietary supplement that has anti-inflammatory, antioxidant, and anti-cancer effects. It suppresses TGF-β (Deng et al., 2009) and iNOS (Gupta et al., 2017) and reduces cellular fibrogenic activity. It also improves the mouth opening and ameliorates the burning sensation in OSF patients (Al-Maweri, 2019). Lycopene is a carotenoid found in watermelon and tomatoes that reduces oxidative damage to lipids, proteins, and DNA. Lycopene ingestion may mitigate oxidative stress in the entire body. A recent clinical trial has shown that oral intake of curcumin improved burning sensation and mouth opening (Saran et al., 2018). Lastly, honey, which is a sweet and viscous food, possesses therapeutic and healing properties due to its anti-inflammatory, antioxidant, and antimicrobial properties. It inhibits the enzyme lipoxygenase, inhibits IL-1, IL-10, and COX-2 expression, scavenges the free radicals, and inhibits the NF-kB signaling pathway. Honey has been used in treating oral diseases such as OSF, halitosis, chemotherapy-induced stomatitis, and radiotherapy-induced oral mucositis (Meo et al., 2017). Table 3 lists all reported conservative OSF therapies and their molecular targets.

**TABLE 3:** Therapeutics approach against OSF. The table enlists the current therapeutic approaches, class of therapy and their molecular targets along with the mechanism.

Name	Therapy class	Molecular target description	References
Hyperbaric Oxygen Treatment	Physical	Promotes fibroblast’s cell death; block TNF-α; TGF-β; preventing collagen synthesis activation	Oscarsson et al. (2017)
Dexamethasone	Drug	Inhibiting activity of anti-inflammatory mediators	Tilakaratne et al. (2016), Sadaksharam and Mahalingam (2017)
Methylprednisolone	Drug	Inhibiting activity of anti-inflammatory mediators	Sadaksharam and Mahalingam (2017)
Betamethasone	Drug	Inhibiting activity of anti-inflammatory mediators	Shetty et al. (2013)
Hyaluronidase	Drug	Hyaluronan Hydrolyzation	Daga et al. (2017)
Chymotrypsin	Drug	Collagen Hydrolyzation	Gupta and Sharma (1988)
Pentoxifylline	Drug	Anti-inflammatory in nature; Prevents TNF-α and leukotriene synthesis	Marques et al. (1999)
Colchicine	Drug	Anti-inflammation; Neutralizing IL-4, IL-6 and TGF-β; Increasing collagenolytic activity	Krishnamoorthy and Khan (2013)
Glabridin	Natural compound	Reducing α-SMA; type I collagen and TGF-β; blocking activity of myofibroblast	Lee et al. (2018)
Butylidenephthalide	Natural compound	Reducing α-SMA; lowering expression of fibronectin and type 1 collagen A1; blocking activity of myofibroblast	Su et al. (2018)
Asiatic acid	Natural compound	Inhibit TGF-β1; collagen 1 type 2 and collagen 3 type 1	Adtani et al. (2017)
Tanshinone	Natural compound	p53 reactivation	Zheng et al. (2018)
Salvianolic acid B with Triamcinolone acetonide	Natural compound	Inhibits COL1A1 and COL3A1 gene transcription; reduces TIMP1/2 expression; Inhibits transcription and release of TGF-β1, CTGF, IL-6 and TNF-α; increases MMP-2 and MMP-9 activity	Jian et al. (2017)
EGCG	Natural compound	Suppresses early growth response-1 (Egr-1) by inhibiting TGF-β1; Supress cellular ROS and inhibits the CTGF and TGM-2 expression	Hsieh et al. (2018)
Aloe vera	Natural compound	Prevents inflammation; and reduce inflammasome formation	Al-Maweri et al. (2019)
Curcumin	Natural compound	p53 inhibition; TGF-β and iNOS inhibition; reduces CTGF	Deng et al. (2009), Gupta et al. (2017)
Lycopene	Natural compound	Works as antioxidants	Gupta et al. (2020)
Honey	Natural compound	Works as anti-oxidant, anti-inflammatory; Inhibits lipoxygenase, IL-1, IL-10, COX-2; NF-kB signaling pathway; scavenge free radicals;	Meo et al. (2017)

### 5.3 Clinical trial

We performed an extensive literature search for ongoing clinical trials of OSF therapies. We found one trial, NCT06871904, where authors are investigating the effect of metformin and pirfenidone in fibrosis modulation in OSF patients. Additionally, authors aim to study the effect of the mechanism underlying the effect of drugs on exosome secretion and protein expression in OSF cell lines. The trial starts in May 2025 and is currently in the recruiting stage. In a separate randomized clinical trial, Batool et al. showed the efficacy of oil extracted from Nigella sativa for treating OSF patients and observed significant results (Batool et al., 2024).

## 6 Computational approaches in OSF

Recent advancements in the computational and bioinformatics approaches, especially in the NGS techniques, have revolutionized the healthcare sector. This holds true in the case of OSF, where computational approaches such as NGS, and AI/ML, have emerged as powerful tools in unraveling the OSF complex molecular landscape. Despite advancements in clinical progression understanding of OSF and its histopathological features, early diagnosis and effective therapeutic targeting are still challenging. Integrating bioinformatics approaches has opened new avenues for the discovery of new biomarkers, molecular profiling, and personalized medicine.

RNA-seq and microarray-based transcriptome quantification have allowed researchers to analyze gene expression changes in OSF patients. For instance, Hu et al. employed oligonucleotide microarray to obtain 716 upregulated and 149 downregulated genes in OSF patients. These genes were enriched for pathways related to immune response, inflammation, EMT, *etc.*, and validated 5 EMT genes, *SFRP4, THBS1, MMP2, ZO-1,* and *CK18*, associated with OSF pathogenesis (Hu et al., 2008). Similarly, Liao et al., based on microarray analyses, reported that overexpression of gene *XRCC5* increased the resistance of GNM cells and promoted cell growth rate (Liao et al., 2016). Recently, Shetty et al. implemented a transcriptomic- and proteomics-based approach and characterized 3 genes, *MMP9, SPARC,* and *ITGA5*, to be associated with OSF pathogenesis (Shetty et al., 2025). Apart from transcriptomics, other omics data types, such as epigenomics and proteomics, are now used by researchers. For example, Kundu et al. perform genome-wide DNA methylation change analysis to observe epigenetic dysregulation of tissues of the oral cavity in the OSF patients. They characterized 3,294 differentially methylated regions and aberrant hypomethylation and high expression of 3 genes, i.e., *FGF13, RPS6KA3,* and *ACSL4* (Kundu et al., 2022). In addition to NGS technologies, other computational approaches such as network pharmacology and molecular docking have been used to uncover novel drugs for treating OSF. For instance, Song et al., in a study, used network pharmacology and molecular docking to characterize 360 OSF-related targets. Functional enrichment revealed processes such as epidermal growth factor receptor (EGFR), advanced glycation end products-receptor (AGE-RAGE), *etc.* A molecular docking study showed that the drug mangiferin binds strongly with targets such as AKT serine kinase 1 (AKT1), tumor necrosis factor (TNF), and several other targets (Ziyi et al., 2024). Similarly, Peng et al. implemented the network pharmacology and docking approach to characterize the predictive targets and pathways of curcuma against OSF and showed ALB and VEGFA as main targets (Peng et al., 2022).

Though limited, several computational resources and *in silico* tools have been developed, addressing the OSF biology. For example, SOFPRO is one such tool, which is developed to perform descriptive epidemiological studies of OSF (Erlewad et al., 2016). Similarly, the areca Nut Database (ANDB) is a web-based repository, developed by Thakur et al. (2020), based on a global survey of literature on AN and associated health effects. Table 4 provides comprehensive information regarding such bioinformatics-based tools, resources, and important datasets generated.

**TABLE 4:** Bioinformatics approach in OSF. The table enlists all the tools, datasets and resources developed in the context of OSF.

Name	Type	Scope/use	Year	References
ANDB	Database	Database compiling information based on literature survey on AN	2020	Thakur et al. (2020)
SOFPRO	Software	Dental informatics based tools for performing descriptive epidemiological studies of OSF	2016	Erlewad et al. (2016)
ORCHID	Database	Database comprising of high-resolution images captured at ×100 magnification.	2024	Chaudhary et al. (2024)
PXD062842	Dataset	Proteomics dataset specific to OSF (circHIPK3 axis)	2025	Zhang et al. (2025)
GSE20170	Dataset	Microarray gene expression profile from 10 OSF patients and pooled normal people	2010	Khan et al. (2011)
GSE64216	Dataset	Microarray gene expression profile from healthy, OSF patients with and without dysplasia.	2016	NA
GSE156669	Dataset	Methylation profiling	2024	Kundu et al. (2022)

## 7 Conclusion and future perspective

OSF cases are rising rapidly at an alarming rate in the world and especially in India. Currently, it is undeniable that OSF possesses the tendency to get transformed into a malignant tumor, and the actual mechanism responsible for it is not yet clear. Understanding the molecular underpinnings of malignant transformation in OSF remains a research priority. Identifying a reliable and low-cost biomarker for timely dysplastic changes and cancer risk will allow timely detection and intervention, significantly improving cancer prevention efforts. Also, it is necessary to establish a robust framework for future management of OSF, as it is expected to shift towards a more proactive, personalized, and resource-sensitive approach.

The key focus will be on developing enhanced and more sensitive diagnostic tools and risk assessment strategies that have the dual property of being accessible and non-invasive. This includes the development of advanced and affordable salivary biomarkers, basic imaging modalities, and running community-level genetic screening programs with the goal of diagnosing and stratifying OSF patients at an early stage to prevent the risk of malignant transformation. Such efforts will allow timely interventions and develop personalized prevention strategies. In parallel, efforts are required to evolve the therapeutic landscape towards more targeted and cost-effective solutions. A major focus should be on developing antifibrotic agents that can be produced locally and are economically viable, for example, small molecule inhibitors against key fibrotic drivers like TGF-β and LOX. In addition, natural compounds with antifibrotic properties can be further explored. Further validation of these therapeutic strategies *via* clinical trials can be done to ensure their efficacy and acceptance in daily clinical practice.

For the comprehensive study of OSF, a favorable animal model is needed, as current models are either arecoline-induced mice or rats. Both models replicate similar pathological conditions and may not imitate the pathogenesis accurately, ultimately restricting the treatment-based research. Hence, there is a need to establish new *in vivo* models that can be used to answer the above questions. Furthermore, there are a few questions that need to be answered for OSF; for instance, type II immune response, which is a chronic inflammatory response, is known to mediate organ fibrosis (liver, lung, heart, *etc.*); however, the role of type II immune response in OSF remains unexplored. Secondly, in OSF pathogenesis, though the role of EMT in epithelial cells has attracted much attention, the role and importance of basal cells, which are pivotal for epithelial cell integrity, have been ignored. Thirdly, the role of human microbiota and its dysregulation in OSF pathogenesis and progression needs comprehensive research.

Regenerative medicine offers a new avenue in treating OSF and restoring oral function in advanced cases. However, due to cost factors and infrastructure constraints, research will increasingly concentrate on simplified regenerative approaches using readily available autologous materials. For example, specific preparation of processed lipoaspirate and low-cost platelet-rich plasma (PRP) for local application. These innovative yet practical methods hold the potential to bring tissue repair within reach of broader patient populations. Further, research will explore strategies such as gene silencing for deregulating fibrotic genes and affordable nanocarrier development for targeted drug delivery. These developments may ultimately revolutionize the treatment paradigm, particularly for individuals with resistant or severe disease. Preventive measures will be equally crucial in alleviating the OSF burden. Community health professionals and localized media outreach will be important for large-scale public health initiatives that will help people stop using ANs and learn about the dangers of OSF. These efforts will be accompanied by stronger advocacy for regulatory controls on the use of AN products to prevent disease initiation at the population level.

Lastly, the shift toward personalized medicine will match treatment plans to each patient’s profiles, disease severity, and the resources available in their area. This personalized strategy will improve clinical outcomes.

## Glossary

OSF: Oral submucosal fibrosis
AN: Areca Nut
WHO: World Health Organization
OSCC: Oral Squamous Cell Carcinoma
HPV: Human Papilloma Virus
EMT: Epithelial-Mesenchymal Transition
NGS: Next-Generation Sequencing
IARC: International Agency for Research on Cancer
CMI: Chronic Mechanical Irritation
ANA: Anti-Nuclear Antibodies
SMA: Smooth Muscle Actin
GPCA: Anti-Gastric Parietal Cell
Hb: Hemoglobin
LOX: Lysyl Oxidase
HLA: Human Leukocyte Antigen
CTLA-4: Cytotoxic T Lymphocyte-Associated Antigen-4
GST: Glutathione S-Transferase
MMPs: Matrix Metalloproteinase
ROS: Reactive Oxygen Species
NO: Nitric oxide
MFBs: Myofibroblasts
TIMPs: Tissue Inhibitors of Metalloproteinases
HIF-1α: Hypoxia-Inducible Factor-1a
VEGF: Vascular Endothelial Growth Factor
TNF-α: Tumor Necrosis Factor-alpha
IHC: Immunohistochemical
PCNA: Proliferating Cell Nuclear Antigen
CYPA: Cyclophilin A
MALDI-IMS: Matrix-Assisted Laser Desorption Imaging Mass Spectrometry
NCOA7: Nuclear Receptor Coactivator 7
E-SOD: Erythrocyte Superoxide Dismutase
GPx: Glutathione Peroxidase
LDH: Lactate Dehydrogenase
MDA: Malondialdehyde
8-OHdG: 8-hydroxy-2-deoxyguanosine
OCT: Optical Coherence Tomography
FTIR: Fourier Transform Infrared Spectroscopy
NADH: Nicotinamide Adenine Dinucleotide
TGF-β1: Transforming Growth Factor-beta 1
HBOT: Hyperbaric Oxygen Therapy
TCM: Traditional Chinese Medicine
EGCG: Epigallocatechin-3-gallate
TGM-2: transglutaminase-2
PRP: Platelet-Rich Plasma
